# Delayed Cutaneous Hypersensitivity Reactions in Patients With Kaposi's Sarcoma

**DOI:** 10.1038/bjc.1974.198

**Published:** 1974-10

**Authors:** J. F. Taylor, J. L. Ziegler

## Abstract

Defects in cellular immunocompetence have been sought in 25 patients with Kaposi's sarcoma. Skin tests with recall antigens, and PHA lymphocyte stimulation *in vitro* showed that efferent delayed hypersensitivity responses are intact in the majority. However, attempted sensitization and subsequent challenge with DNCB demonstrated that the afferent limb of the responses was impaired in some patients. This did not appear to be related to the morphology of the tumour or to prognosis.

Tumour specific reactions were demonstrated both *in vivo* and *in vitro* and these correlated significantly with the morphology and histology. The interpretation of the results for an individual is confounded by the multiplicity of factors influencing the outcome in a particular patient.


Br. J. Cancer (1974) **30**, 312

### DELAYED CUTANEOUS HYPERSENSITIVITY REACTIONS IN PATIENTS WITH KAPOSI'S SARCOMA

J. F. TAYLOR AND J. L. ZIEGLER

*From the Department of Orthopaedics, Box 147, Liverpool L69 3BX, U.K. and the Paediatric Oncology Branch, N.C.I., N.I.H., Bethesda, Maryland, 20014, U.S.A.*

Received 13 May 1974. Accepted 20 June 1974

**Summary.—Defects in cellular immunocompetence have been sought in 25 patients with Kaposi's sarcoma. Skin tests with recall antigens, and PHA lymphocyte stimulation *in vitro* showed that efferent delayed hypersensitivity responses are intact in the majority. However, attempted sensitization and subsequent challenge with DNCB demonstrated that the afferent limb of the responses was impaired in some patients. This did not appear to be related to the morphology of the tumour or to prognosis.**

**Tumour specific reactions were demonstrated both *in vivo* and *in vitro* and these correlated significantly with the morphology and histology. The interpretation of the results for an individual is confounded by the multiplicity of factors influencing the outcome in a particular patient.**

THE PIGMENTED haemangiosarcoma first described by Kaposi in 1872 is a rare malignant tumour, more prevalent in equatorial Africa than in temperate areas of the world. Though the restricted geographical distribution may indicate an inherited susceptibility to the tumour, it is of interest that herpesviruses have been located in long-term cultures of the sarcoma. Their relevance to the aetiology is as yet uncertain (Giraldo, Beth and Haguenau, 1972). In adults, the disease is primarily cutaneous but in children it involves the lymph nodes, skin lesions being uncommon (U.I.C.C., 1962).

The natural history of the disease is variable and correlates with the gross morphology and histology of the skin tumour (Taylor *et al.*, 1971*a*). Small nodules are relatively benign and may show spontaneous regression and disappear (Lothe, 1963). Florid tumours are exophytic and lack skin cover whereas infiltrative lesions penetrate deeper tissues and are associated with dense fibrosis, but both are more aggressive than nodular disease. The lymphadenopathic form is disseminated and rapidly fatal.

Histologically, the diagnosis depends on the recognition of spindle cells, vascular slits and vascular channels lined by endolethial cells. In nodular and adenopathic disease these elements are present in equal proportions (mixed cell pattern). In florid and infiltrative lesions, sheaves of spindle cells predominate (monocellular pattern). However, occasional tumours show cellular pleomorphism and frequent mitoses (anaplastic pattern) (Taylor *et al.*, 1971*a*). Lymphocytes are commonly seen in nodular and infiltrative lesions, both of which are associated with a fibrotic reaction.

Immunological studies of the sarcoma in Uganda (Master *et al.*, 1970) demonstrated normal humoral antibody responses and immunoglobin levels in all patients. However, a striking impairment of the delayed cutaneous hypersensitivity response to dinitrochlorobenzene (DNCB) was noted in patients with " malignant " (florid) tumours. *In vitro* studies (Taylor *et al.*, 1971*b*) also showed a correlation between the gross tumour morphology and the capacity of lymphocytes to be stimulated in culture by phytohaemagglutinin (PHA) or tumour cells. Whereas transformation in response to PHA was depressed in lymphocytes from patients with florid tumours, a positive response to both PHA and to mixed culture with autologous tumour cells was noted in lymphocytes from patients with nodular or infiltrative tumours. These findings led to the concept that the favourable prognosis in patients with nodular disease is associated with a delayed hypersensitivity response to tumour, which is lacking in those with florid disease.

### MATERIALS AND METHODS

Patients admitted to the Uganda Cancer Institute or Mulago Hospital between September 1969 and July 1970 with a clinical diagnosis of Kaposi's sarcoma were all potential participants in this study. Those not studied withheld consent for the tests or lacked sufficient tumour tissue for both a diagnostic biopsy and preparation of cell extracts. None had had chemotherapy in the previous 2 years. On admission, the patients underwent an extensive clinical and radiological evaluation, and each had a full blood count. The cutaneous tumours were classified as nodular, florid or infiltrative, and also according to the Rules for Tumour Classification (U.I.C.C., 1968). For subsequent therapy, the patients were randomized as described by Vogel *et al.* (1971). The clinical and research investigations were all approved by a committee at Makerere University Medical School.

*Preparation of cell extracts.*—Sterile techniques were used throughout. Tumour tissue was obtained at the time of diagnostic biopsy. Where possible, an area of tumour-free skin was excised for preparation of the control extract, obtained as described by Taylor *et al.* (1971*b*) but lymphocytes were used if sufficient skin was not available. The method used to prepare the extracts has been described in detail by Fass, Herberman and Zeigler (1970). Cell suspensions were obtained by mincing the tissue and cells disrupted by freezing, and subsequent exposure to saline solutions of decreasing tonicity. After each hypotonic lysis, the suspension was centrifuged and the supernatant fluid collected and pooled. This was then concentrated over a 36-h period by pressure dialysis at 4°C to a final volume of 1 ml. Following preparation, the protein concentration of the extracts was determined by the method of Lowry *et al.* (1951). The extracts were made up to 3 protein concentrations (1 mg/ml, 0·5 mg/ml and 0·1 mg/ml) divided into 1 ml aliquots in tuberculin syringes and stored at −70°C until testing. The preparations were checked for bacteriological contamination by blood agar culture before testing.

*Administration and interpretation of the skin tests.*—Initial skin tests were undertaken before chemotherapy. 0·1 ml of the following antigens was injected intradermally in that area of the back between the scapulae: autologous tumour extract (ATE) and control skin extract (ASE), intermediate strength tuberculin (Parke, Davis, Detroit), mumps skin test antigen (Eli Lilly & Co., Indiana), *Candida albicans* (as Dermatophytin "0" 1 : 100, Hollister Steir Laboratories, Washington), brucella antigen (as Brucellergen Protein Nucleate, Merke, Sharp and Dohme, Pennsylvania) and streptokinase–streptodornase (Lederle, New York). Skin tests were read at 24 and 48 h by 2 individuals, one of whom was unaware of the arrangement of the injections. A positive test was defined as one having induration of at least 5 mm in diameter.

Skin punch biopsies were performed on all positive tumour extract skin tests and corresponding control sites. Histological sections from these biopsies were reported under code by an independent observer. Perivascular mononuclear cell accumulations at the site of ATE injection and not ASE, were required for the test to be recorded as positive.

Delayed hypersensitivity to DCNB (1-chloro-2,4-dinitrobenzene, Eastman Chemicals, New York) was tested using a 2000 $\mu$g sensitizing dose, which was allowed to evaporate within a 2 cm diameter polyethylene ring applied to the right forearm. The area had been previously cleaned with acetone and was subsequently covered by an occlusive dressing. Fourteen days later, a 100 $\mu$g challenge was applied to another site. This was examined at 48 h and counted as positive if induration, vesicles and bullae were present.

*Lymphocyte transformation by PHA and tumour cells.*—Lymphocytes were cultured from a sub-sample of the patients selected only by availability of sufficient blood or

TABLE I.—*Patient Characteristics, Type of Tumour and Immunological Test Results*

| Patient | Age | Sex | Duration of symptoms in months | Tumour histology | Bacterial antigens, no. of sites showing positive reaction | Response to DNCB | Source of tissue extracts | Reaction to ATE | Lymphocyte transformation ratio: PHA/control | Lymphocyte transformation ratio: Tumour cell/control | Patients' response to treatment |
|---|---|---|---|---|---|---|---|---|---|---|---|
| *Nodular* | | | | | | | | | | | |
| 2 | 60 | M | 18 | Monocellular | 2 | − | S | − | 55 | 2·8 | B |
| 4 | 28 | M | 84 | Mixed cell | 2 | − | L | + | ND | ND | B |
| 6 | 70 | M | 30 | Mixed cell | 2 | + | S | + | 8·2 | 0·9 | B |
| 12 | 42 | M | 14 | Mixed cell | 4 | + | S | + | ND | ND | B |
| 14 | 35 | M | 12 | Mixed cell | 2 | − | S | + | 54 | ND | B |
| 15 | 53 | M | 60 | Mixed cell | 3 | + | L | + | ND | ND | B |
| 16 | 41 | M | 48 | Mixed cell | 1 | + | S | − | 5·8 | 1·1 | B |
| 17 | 28 | M | 36 | Mixed cell | 1 | − | S | − | ND | ND | B |
| *Florid* | | | | | | | | | | | |
| 3 | 40 | M | 2 | Anaplastic | 0 | − | L | − | 3·7 | 0·9 | D |
| 5 | 43 | M | 6 | Monocellular | 3 | + | S | − | ND | 0·8 | B |
| 7 | 55 | M | 24 | Anaplastic | 4 | + | S | ND | 31 | ND | B |
| 8 | 20 | M | 2 | Mixed cell | 1 | + | L | − | 0·2 | 0·3 | B |
| 9 | 35 | M | 48 | Monocellular | 1 | − | S | + | 0·9* | 0·7* | B |
| 13 | 42 | M | 36 | Anaplastic | 3 | − | S | − | 2·2 | 0·7 | W |
| 18 | 26 | F | 2 | Mixed cell | 1 | − | L | − | ND | ND | D |
| 20 | 70 | M | 24 | Monocellular | ND | + | S | − | ND | ND | B |
| 22 | 50 | M | 9 | Mixed cell | 3 | + | L | − | 18·5 | 3·1 | (D)* |
| 23 | 50 | M | 12 | Monocellular | 2 | + | S | − | 1·5 | 0·8 | B |
| *Infiltrative* | | | | | | | | | | | |
| 1 | 50 | M | 36 | Monocellular | 1 | − | S | − | 11·6 | 0·9 | NC |
| 11 | 60 | M | 20 | Monocellular | 1 | − | L | − | 1·2 | 2·2 | NC |
| 19 | 50 | M | 240 | Mixed cell | 2 | − | S | + | 3·3 | 0·6 | B |
| 24 | 35 | M | 132 | Monocellular | 2 | + | S | + | 4·3 | 1·9 | B |
| *Lymphadenopathic* | | | | | | | | | | | |
| 10 | 15 | F | 9 | Mixed cell | 2 | − | L | − | 20·8 | 1·0 | B |
| 21 | 2 | M | 0·3 | Mixed cell | 1 | − | S | − | ND | ND | B |
| 25 | 4 | M | 0·2 | Mixed cell | 2 | + | S | − | ND | ND | D |

S = skin L = lymphocytes ND = not done B = better NC = no change D = died W = worse * see text.

tumour tissue, the techniques being those fully described by Taylor *et al.* (1971*b*). The cells were washed once with medium RPMI 1640 (Gibco, N.Y., U.S.A.) and then resuspended in this medium supplemented with 20% gammaglobulin-free foetal calf serum. Two ml of the suspension, containing $2 \times 10^6$ mononuclear cells was incubated with PHA, autologous tumour cells, or control lymphocytes which had been previously inhibited by mitomycin C. Lymphocyte transformation was estimated by measuring the uptake of iodo-uridine ($^{125}$I) into lymphoblasts, using a gamma spectrometer. The results (Table I) are expressed as the ratio of iodo-uridine in stimulated cultures: uptake in control tubes. In the previous work a tumour : control uptake ratio of greater than 1·5 was regarded as positive evidence of transformation induced by tumour cells.

### RESULTS

The clinical features of the 25 patients and the results of the immunological evaluation are shown in Table I.

There was no correlation between possible responses to these tests and classifications of the tumours, based on U.I.C.C. rules. The table is therefore subdivided on the basis of gross tumour morphology.

Of 25 patients skin tested with bacterial antigens one or more positive recall responses were seen in all but one (patient No. 3). This man had an anaplastic, florid tumour. Negative responses were obtained to all the tests and he died of his tumour, despite intensive chemotherapy.

The results of dinotrochlorobenzene testing are shown in Table II, 12 of 25 patients having positive responses. This proportion of positive : negative responses was seen in patients with both florid and nodular tumours, the few patients with infiltrative or lymphadenopathic forms responding less frequently. Of 9 patients with less than 2 positive recall responses, only 2 had a positive DNCB reaction, whereas of 15 patients with 2 or more positive recall responses, 9 had positive DNCB tests ($0{\cdot}1 < P < 0{\cdot}05$).

No significant correlation was noted between positive DNCB skin tests and the skin response to ATE or lymphocyte transformation induced by PHA or tumour cells. Ten of those with positive responses and 8 with negative DNCB skin tests improved with chemotherapy. Of the 2 who died, one (No. 22) died of a cardiovascular collapse unrelated to his disease or therapy.

Bacteria were cultured from a number of the primary tumours, but the ATE and ASE were sterile in the 25 patients listed in Table I. In a further 2 patients, blood agar culture revealed bacterial contamination and these were excluded from the study. No immediate skin reactions were noted, the induration being maximal at 48 h. Positive responses were maximal at the 1·0 mg/ml injection site, with the exception of that of patient No. 14, in whom the 0·5 mg/ml site had the greater diameter.

Perivascular mononuclear infiltrates were observed in all of the positive ATE skin test sites, with the exception of patient No. 13 in whom a micro-abscess was demonstrated, and the test counted as negative. Control (ASE or ALE) sites were clinically negative, with the exception of patient No. 19. However, the biopsy

TABLE II.—*Incidence of Delayed Cutaneous Hypersensitivity to DNCB and Autologous Tumour Extract (ATE) in Patients with Different Gross Tumour Morphology*

| Type of tumour | DNCB No. positive per no. tested | DNCB % positive | ATE No. positive per no. tested | ATE % positive |
|---|---|---|---|---|
| Nodular | 4/8 | 50 | 5/8 | 63 |
| Infiltrative | 1/4 | 25 | 2/4 | 50 |
| Florid | 6/10 | 60 | 1/9 | 11 |
| Lymphadenopathic | 1/3 | 33 | 0/3 | 0 |
| Total | 12/25 | | 8/24 | |

TABLE III.—*Correlation of the Response to DNCB and Tumour Extract with Tumour Histology*

| | DNCB | | ATE | |
|---|---|---|---|---|
| Tumour histology | No. positive per no. tested | % positive | No. positive per no. tested | % positive |
| Mixed cell | 7/14 | 50 | 6/14 | 43 |
| Monocellular | 4/8 | 63 | 2/8 | 25 |
| Anaplastic | 1/3 | 30 | 0/2 | 0 |
| Total | 12/25 | | 8/24 | |

revealed no mononuclear infiltration and the response to ATE has been regarded as positive. Because of tumour induced lymphocyte transformation in culture, the skin test sites were also biopsied in patient No. 22. Both showed perivascular mononuclear infiltrate, more marked at the site of ATE.

Eight of 24 patients showed positive results to ATE (Table II). Seven of these were in patients with nodular or infiltrative tumours but the frequency was significantly lower in those with florid or lymphadenopathic lesions ($P < 0{\cdot}025$, Fischer's exact test). There was also correlation with the histology of the tumour (Table III), the incidence of positive results being greatest in those whose tumours had a mixed cell pattern. The duration of symptoms was greater than a year in those with positive response to ATE and all improved after chemotherapy. No correlation is seen between positive responses to ATE skin tests and *in vitro* tumour induced transformation. However, in 4 of those with positive ATE responses, the tumour nodules were too small to provide sufficient cells for both tests.

Only one patient (No. 9) with a florid tumour showed a positive response to ATE and on admission lymphocyte transformation was negative. His tumour responded completely to chemotherapy and he was re-tested on remission. The ATE skin test was positive, as was PHA induced transformation (ratio 129) and transformation induced by the ATE used for skin test (ratio 1·6).

### DISCUSSION

Kaposi's sarcoma, in the majority of adult Africans, is a distinctive clinical entity but the tumour morphology may be variable. Tedeschi (1958) believed that a tumour changed its appearance as the disease progressed, but Reynolds, Winkelmann and Soule (1965) described many cutaneous forms and implied that each retained its integrity during the course of the disease. This concept was confirmed by Taylor *et al.* (1971*a*), who found that differing histological patterns correlated with the tumour morphology (Table IV). Nevertheless, histological differences were inadequate to explain the variable outcome. "Benign" nodules and the fatal lymphadenopathic form of the disease both have a mixed cell histopathology. The differing prognoses could be explained by variation either of tumour cell antigens or of the host response.

In the present study, the majority of patients were able to exhibit delayed hypersensitivity recall responses when challenged with bacterial antigens. Only one patient with an advanced florid tumour was unresponsive to all antigens and to DNCB sensitization. This contrasts with the findings of Solowey and Rappaport (1965) that of 150 patients with other cancers, 110 were anergic to the test materials. The responses to these intradermal injections, which test only the effector mechanism, did not correlate with the morphology of Kaposi's sarcoma or the subsequent clinical course.

The reactions to DNCB were related to tumour morphology and reflected to

TABLE IV.—*Summary of Types of Kaposi's Sarcoma*

| Clinical type | Behaviour | Age group (years) | Typical histopathology | Response to chemotherapy[1] |
|---|---|---|---|---|
| Nodular | Indolent | Over 25 | Mixed cell | Remission |
| Florid | Locally aggressive | Over 25 | Monocellular[2] | Remission[3] |
| Infiltrative | Locally aggressive | Over 25 | Monocellular | Refractory |
| Lymphadenopathic | Disseminated | Under 25 | Mixed cell | Refractory |

[1] From Vogel *et al* (1971).
[2] Anaplastic histopathology may also be seen in florid tumours.
[3] Anaplastic variants are usually refractory to treatment.

some degree the extent of reactivity to recall antigens. As DNCB testing involves both the afferent and effector immune processes, it appears that non-reactive patients lack the capacity to be sensitized to the synthetic antigen. It is not known whether such a defect leads to, or results from, development of the tumour, and Kaposi's sarcoma has been reported following immunosuppressive therapy (Siegel *et al.*, 1969). The correlation between tumour morphology and DNCB sensitization was not as clearly defined, as in a series of patients reported previously by Master *et al.* (1970) in which all patients with nodular disease reacted to DNCB. However, the morphology of the principal tumour was classified differently in the 2 series and this may account for some discrepancy. The 4 negative responses to DNCB in adults with nodular disease may have been the result of excessively critical criteria used in defining a positive response in the present study, as lymphocyte transformation in response to PHA was demonstrated in 2 of these patients.

The little information available from the *in vitro* studies with PHA confirms the recall tests with bacterial antigens by demonstrating the presence of competent circulating lymphocytes in patients of each clinical group. However, in lymphocytes from some patients, the response to PHA is considerably impaired, and similar results have been reported in Hungarian patients by Dobozy *et al.* (1973).

The skin reactions evoked by autologous tumour extract (ATE) demonstrated the clinical and histological features of delayed hypersensitivity reactions. Negative reactions to ATE did not result from nonspecific cutaneous anergy, as the majority of patients with negative reactions had positive responses to recall bacterial antigens and/or DNCB. The antigens which evoked the positive responses to ATE appear to be tumour specific, as negative responses were recorded at the site of control injections. The concept that skin reactivity to ATE reflects the response to tumour antigen, and not to contaminating bacterial products, is further supported in the present work by the greater frequency of positive responses in patients with nodular and infiltrative tumours compared with other types. While bacterial antigens would be expected in greater concentration in fungating lesions, the frequency of positive responses to ATE were invariably associated with improvement after therapy (Table I) but a negative test was not predictive.

This study adds Kaposi's sarcoma to the growing list of human tumours in which it is possible to demonstrate specific cell mediated immunity by cutaneous reactivity to ATE. The majority of these responses were evident in patients with indolent tumours, and were invariably associated with favourable responses of the tumour to chemotherapy. However, occasional responses to ATE were seen in patients with florid or infiltrative tumours.

This implies that the lack of reactivity in the majority of patients with these aggressive tumours is not entirely due to lack of antigens. It may, however, reflect the interaction of humeral and cellular immune mechanisms, a blocking factor protecting the tumour from the cellular immune response. A search for such antibodies would be a logical extension of this study of patients with Kaposi's sarcoma.

The authors wish to thank Mrs V. MacMillan and Mr. L. Sebwami for preparing the extracts; Drs A. Bluming, D. Büttner, L. Fass, R. B. Herberman and C. Vogel for assistance with this study and for supplying the bacterial antigens; Dr F. Deinhardt and Professor S. Kyalwazi for additional advice and Dr A. Templeton for reviewing the histological sections.

This work was supported by the Cancer Research Campaign (B.E.C.C.), by Contracts No. 47-67-1343 and No. 43-62-179 of the National Cancer Institute, National Institutes of Health, Bethesda, Maryland, and by Makerere University.

### REFERENCES

Dobozy, A., Husz, S., Hunyadi, J., Berkko, G. & Simon, N. (1973) Immune Deficiencies and Kaposi's Sarcoma. *Lancet*, ii, 625.

Fass, L., Herberman, R. B. & Ziegler, J. (1970) Delayed Cutaneous Hypersensitivity Reactions to Autologous Extracts of Burkitt Lymphoma Cells. *New Engl. J. Med.*, **282**, 776.

Giraldo, G., Beth, E. & Haguenau, F. (1972) Herpes-type Virus Particles in Tissue Cultures of Karposi's Sarcoma. *J. natn. Cancer Inst.*, **49**, 1509.

Kaposi, M. (1872) Idiopathisches multiples Pigmentsarkom der haut. *Arch. Derm. Syph.*, **4**, 265.

Lothe, F. (1963) Kaposi's Saroma in Ugandan Africans. *Acta path. microbiol. scand. Suppl.*, **161**, 1.

Lowry, O. H., Rosenbrough, N. J., Farr, A. L. & Randall, A. J. (1951) Protein Measured with the Folin Phenol Reagent. *J. biol. Chem.*, **193**, 265.

Master, S. P., Taylor, J. F., Kyalwazi, S. K. & Ziegler, J. L. (1970) Immunological Studies in Kaposi's Sarcoma. *Br. med. J.*, i, 600.

Reynolds, W. A., Winklemann, R. K. & Soule, E. H. (1965) Kaposi's Sarcoma. A Clinicopathologic Study with Particular Reference to its Relationship to the Reticuloendothelial System. *Medicine, Baltimore*, **44**, 419.

Siegel, J. H., Janis, R., Atpor, J. C., Schutte, H., Robins, L. & Blaufox, M. D. (1969) Disseminated Visceral Kaposi's Sarcoma. *J. Am. med. Ass.*, **207**, 1493.

Solowey, A. C. & Rappaport, F. T. (1965) Immunological Responses in Cancer Patients. *Surg. Gynec. Obstet.*, **14**, 756.

Taylor, J. F., Templeton, A. C., Vogel, C. L., Ziegler, J. L. & Kyalwazi, S. K. (1971*a*) Kaposi's Sarcoma in Uganda. *Int. J. Cancer*, **8**, 127.

Taylor, J. F., Junge, U., Wolfe, L., Deinhardt, F. & Kyalwazi, S. K. (1971*b*) Lymphocyte Transformation in Patients with Kaposi's Sarcoma. *Int. J. Cancer*, **8**, 468.

Tedeschi, C. G. (1958) Some Considerations Concerning the Nature of the So-called Sarcoma of Kaposi. *A.M.A. Archs Path.*, **66**, 65.

U.I.C.C. (1962) Symposium on Kaposi's Sarcoma. *Acta Un. Int. Cancr.*, **18**.

U.I.C.C. (1968) *T.N.M. Classification of Malignant Tumours.*

Vogel, C. L., Templeton, C. J., Templeton, A. C., Taylor, J. F. & Kyalwazi, S. K. (1971) Treatment of Kaposi's Sarcoma with Actinomycin D and Cyclophosphamide. *Int. J. Cancer*, **8**, 136.


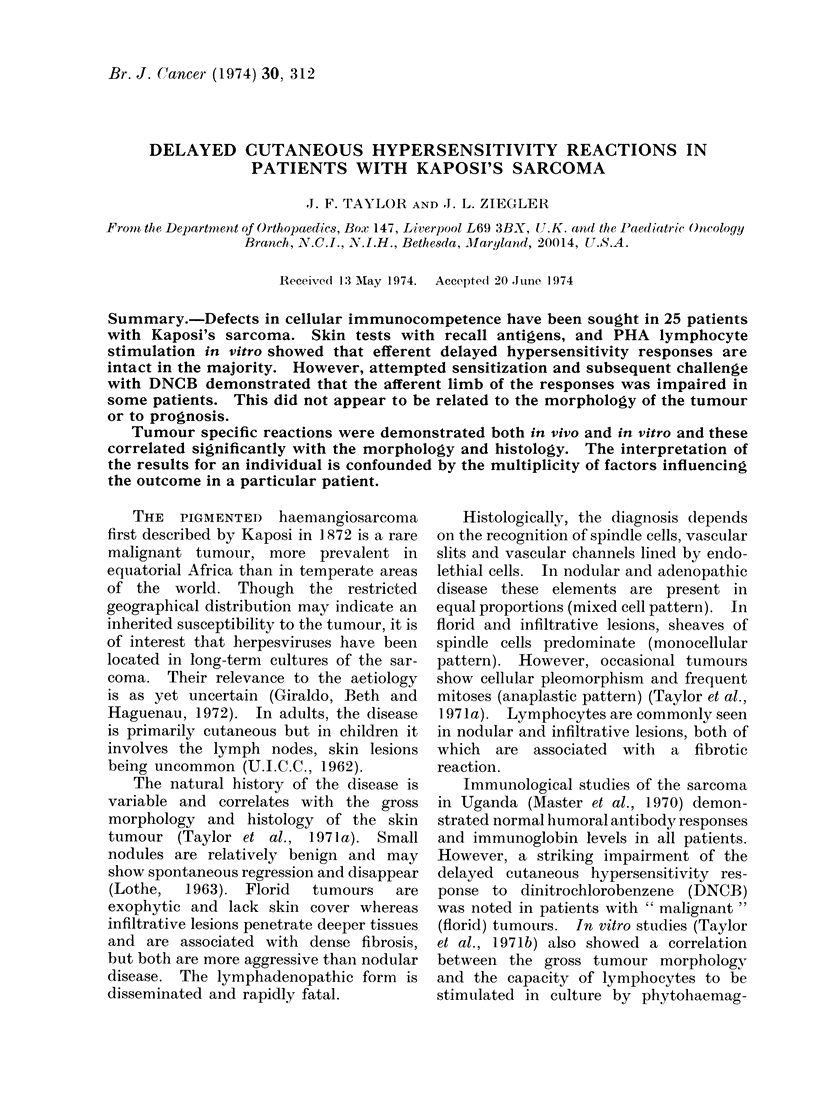

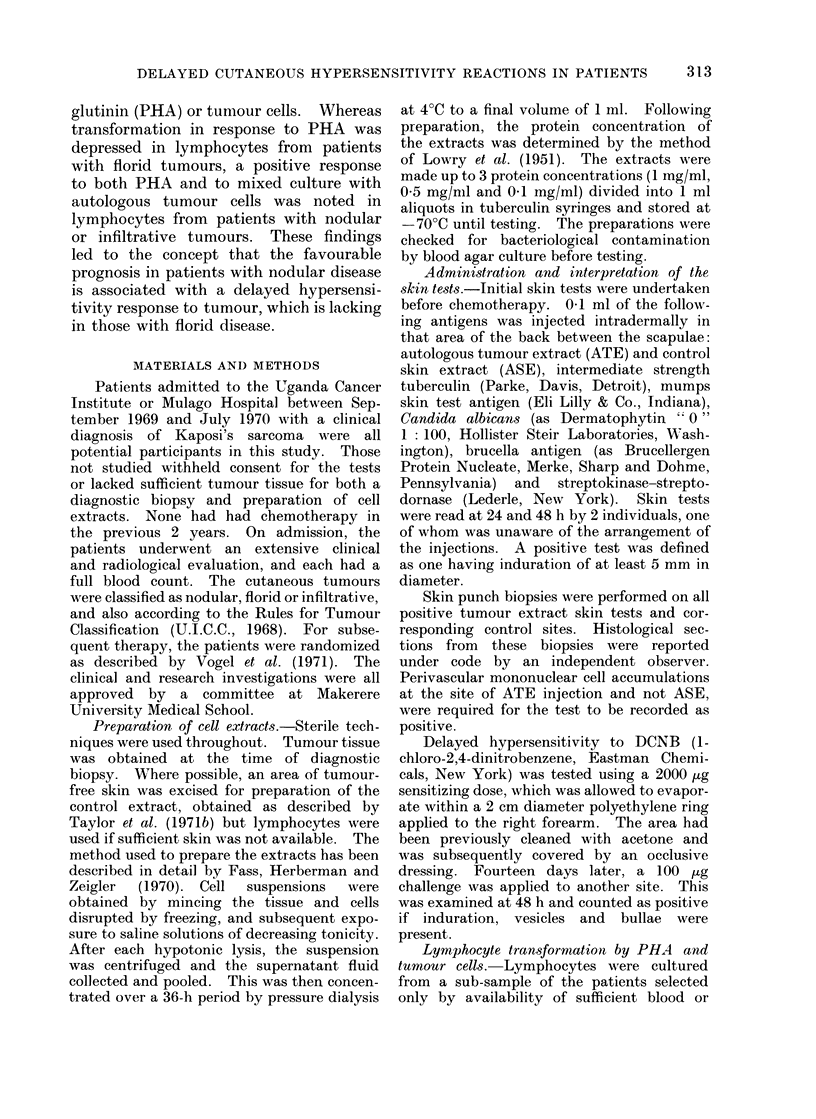

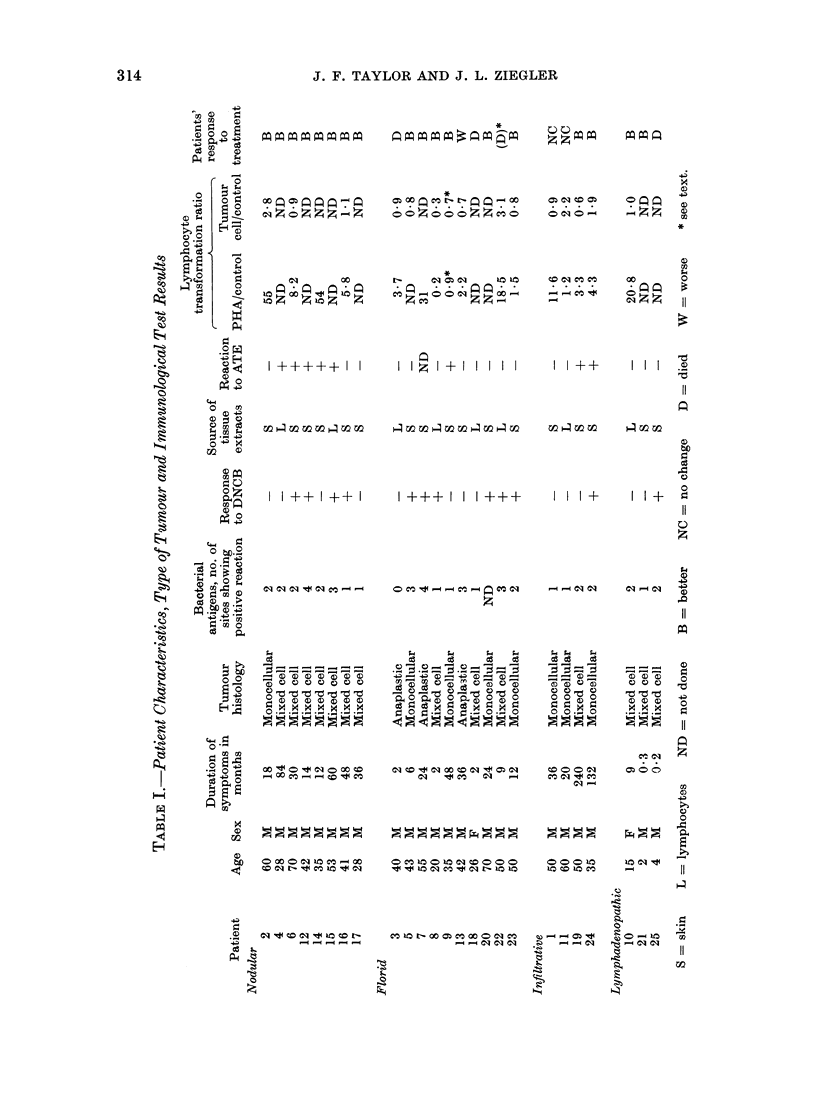

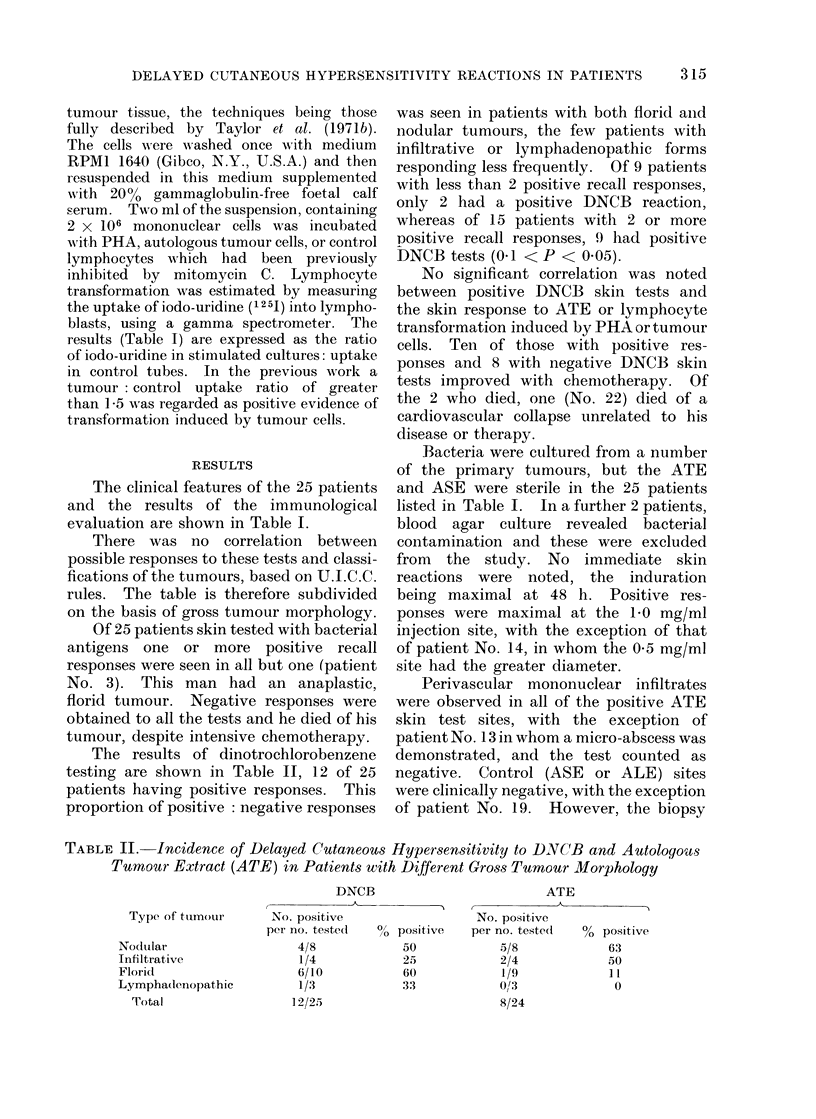

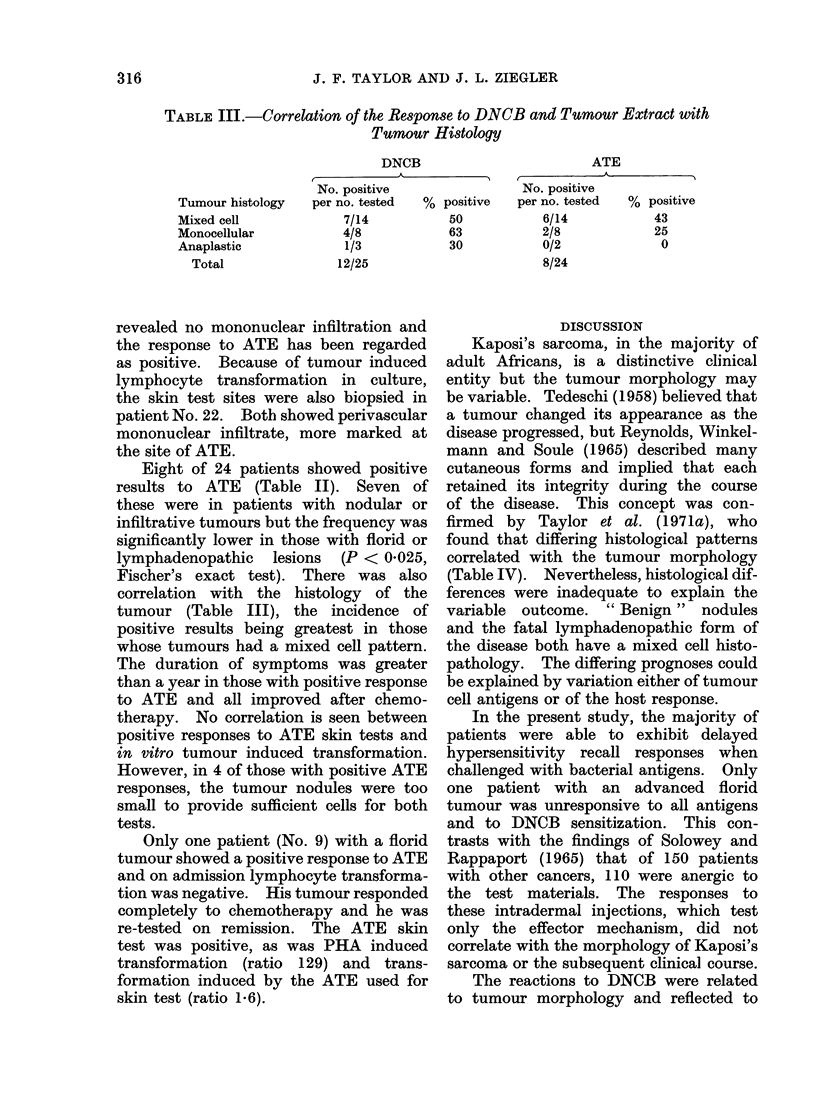

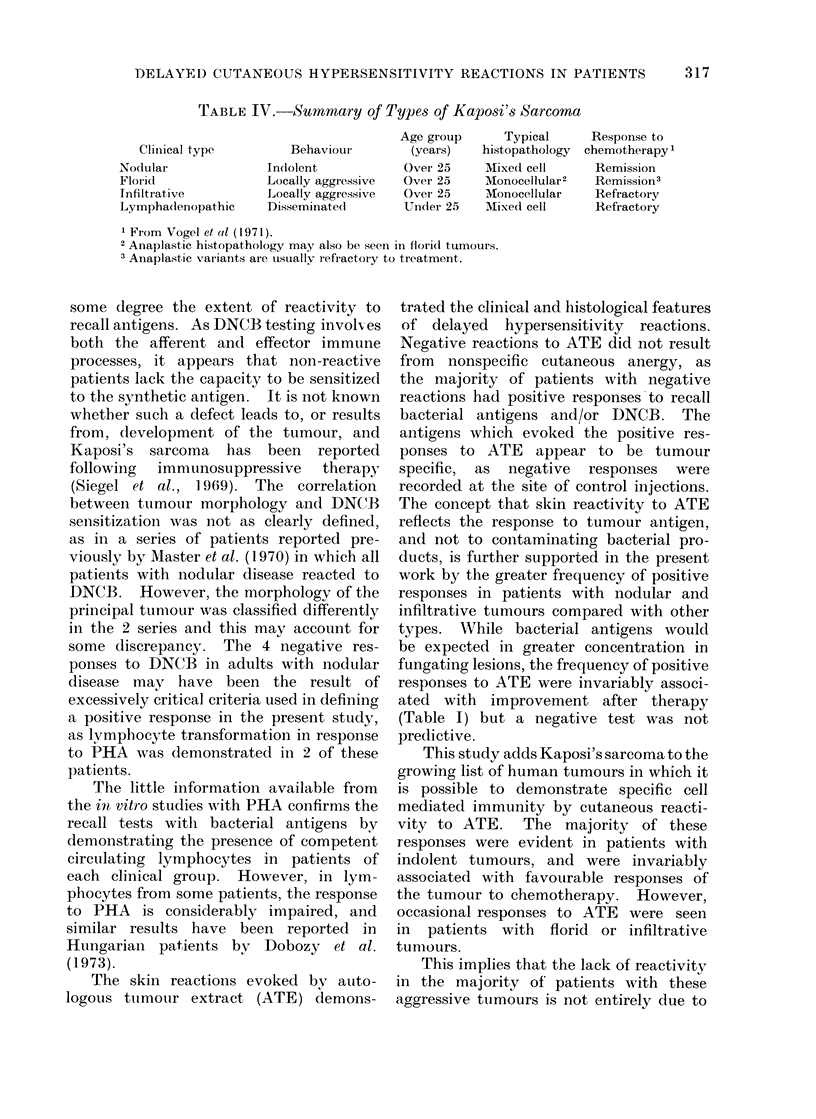

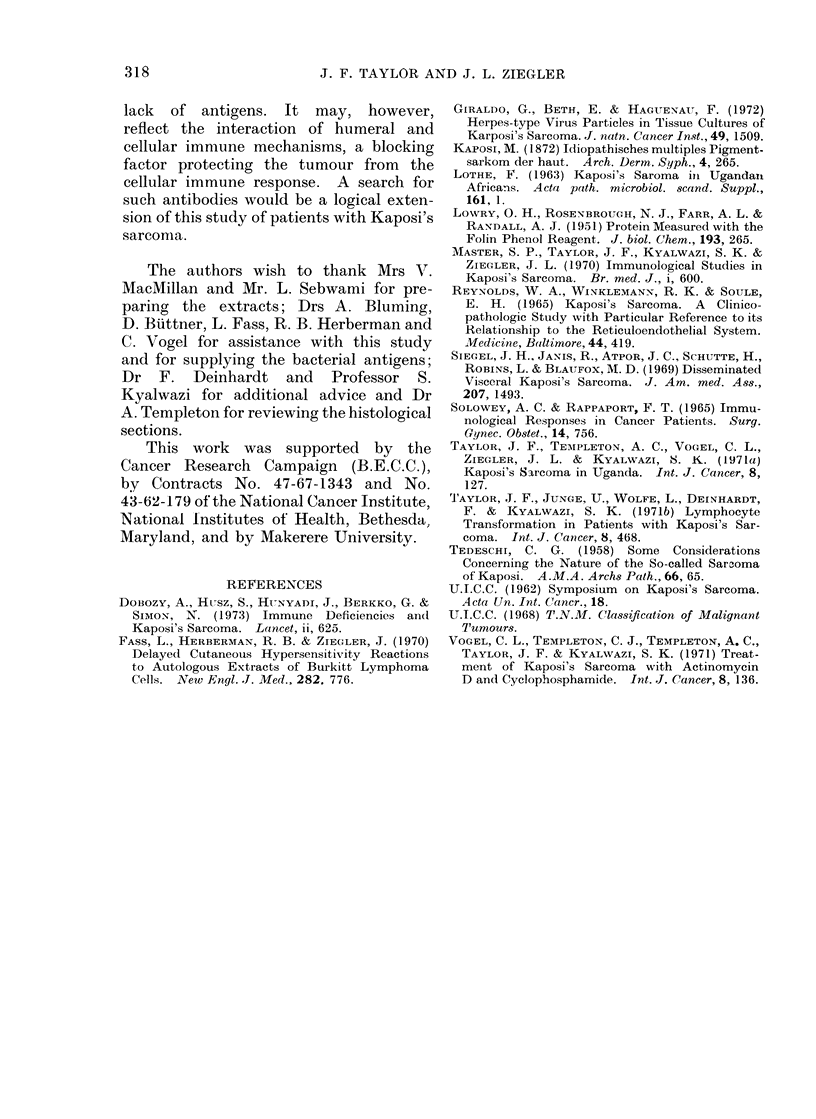


## References

[OCR_00741] Dobozy A., Husz S., Hunyadi J., Berkó G., Simon N. (1973). Immune deficiencies and Kaposi's sarcoma.. Lancet.

[OCR_00746] Fass L., Herberman R. B., Ziegler J. (1970). Delayed cutaneous hypersensitivity reactions to autologous extracts of Burkitt-lymphoma cells.. N Engl J Med.

[OCR_00752] Giraldo G., Beth E., Haguenau F. (1972). Herpes-type virus particles in tissue culture of Kaposi's sarcoma from different geographic regions.. J Natl Cancer Inst.

[OCR_00765] LOWRY O. H., ROSEBROUGH N. J., FARR A. L., RANDALL R. J. (1951). Protein measurement with the Folin phenol reagent.. J Biol Chem.

[OCR_00770] Master S. P., Taylor J. F., Kyalwazi S. K., Ziegler J. L. (1970). Immunological studies in Kaposi's sarcoma in Uganda.. Br Med J.

[OCR_00775] Reynolds W. A., Winkelmann R. K., Soule E. H. (1965). Kaposi's sarcoma: a clinicopathologic study with particular reference to its relationship to the reticuloendothelial system.. Medicine (Baltimore).

[OCR_00782] Siegel J. H., Janis R., Alper J. C., Schutte H., Robbins L., Blaufox M. D. (1969). Disseminated visceral Kaposi's sarcoma. Appearance after human renal homograft operation.. JAMA.

[OCR_00788] Solowey A. C., Rapaport F. T. (1965). Immunologic responses in cancer patients.. Surg Gynecol Obstet.

[OCR_00799] Taylor J. F., Junge U., Wolff L., Deinhardt F., Kyalwazi S. K. (1971). Lymphocyte transformation in patients with Kaposi's sarcoma.. Int J Cancer.

[OCR_00818] Vogel C. L., Templeton C. J., Templeton A. C., Taylor J. F., Kyalwazi S. K. (1971). Treatment of Kaposi's sarcoma with actinomycin-D and cyclophosphamide: results of a randomized clinical trial.. Int J Cancer.

